# A Median Analysis of Factors Influencing Body Fatness in Urban School-Age Children in Cameroon

**DOI:** 10.1155/2019/1856069

**Published:** 2019-02-03

**Authors:** Loveline L. Niba, Paul B. Itor, Yemele K. Sibelle Aurelie, Foba M. Singam, Emmanuel A. Tange, Mary B. Atanga, Lifoter K. Navti

**Affiliations:** ^1^Department of Biochemistry, Catholic University of Cameroon (CATUC), P.O. Box 782, Bamenda, Cameroon; ^2^Nutrition and Health Research Group (NHRG), Bamenda, Cameroon; ^3^Department of Microbiology, Catholic University of Cameroon (CATUC), P. O. Box 782, Bamenda, Cameroon; ^4^Department of Public Health and Hygiene, University of Buea, P.O. Box 63, Buea, Cameroon; ^5^Department of Nursing and Midwifery, University of Bamenda, P.O. Box 39, Bambili, Bamenda, Cameroon; ^6^Department of Food Science and Technology, Catholic University of Cameroon (CATUC), P.O. Box 782, Bamenda, Cameroon

## Abstract

**Background:**

Childhood overweight/obesity is a fast growing public health problem in developing countries. The adverse health consequences of obesity have been attributed to higher body fat levels and this has drawn overwhelming attention towards more accurate assessment of body fat. The goal of this study is to evaluate the relationships between selected behavioral factors and percentage body fat (%BF) estimated using bioelectrical impedance analysis in school-age children.

**Methods:**

A cross-sectional analysis was carried out in randomly selected 6- to 11-year-old children (507 boys and 501 girls). Percentage body fat was assessed using bioelectrical impedance analysis. The behavioral factors were reported by parents using a structured questionnaire. Multiple quantile regression analysis was used to evaluate the relationship between the selected behavioral factors and %BF.

**Results:**

With quantile regression, the daily consumption of fruits and vegetables, daily breakfast consumption, and high physical activity (>4–7 times/week) were significantly (*p* < 0.001) associated with a 4.95, 3.29, and 3.66 decrease in median %BF, respectively. Also, consumption of snacks (>3 times a day) (*p* < 0.001), high sedentary lifestyle (>3–6 hours/day) (*p* < 0.001), and motorization to school (*p* < 0.005) significantly increased the median %BF by 3.69, 3.01, and 1.39, respectively. The largest changes in median %BF were observed in girls.

**Conclusions:**

Efforts are needed using longitudinal studies to clarify the effects of these behavioral factors on %BF in different regions and ethnic groups of Cameroon and also to assess whether any observed differences are of clinical relevance.

## 1. Introduction

The prevalence of overweight/obesity worldwide has almost tripled over the last four decades [1]. A recent report has estimated that if the current trends of overweight/obesity among 5- to 17-year-old children are not abated with effective intervention policies, then the global numbers of overweight and obese children may reach 268 million and 90 million, respectively, by 2025 [2]. A recent review that included sub-Saharan African (SSA) countries has also indicated a transition to higher numbers of overweight/obese school-age children, which is similar to trends observed in some developed countries. However, the weighted averages of overweight/obesity were lower when compared with those of some developed countries [3].

In Cameroon, data on overweight/obesity in school-age children are available mostly from urban settings in some administrative regions of the country. For instance, recent reports show that the prevalence of overweight/obesity in the Littoral Region among 3- to 13-year-old and 8- to 15-year-old school children is 12.5% [4] and 14.4% [5], respectively, while in the North West Region, the prevalence among 5- to 12-year old school children is 17.6% [6].

Overweight/obesity has been associated with adverse health consequences, including unfavorable blood pressure levels and cardiovascular disease risk [7, 8] in children and increased risk of type 2 diabetes in adulthood. For example, a pooled analysis has indicated that increase in body fat is contributing to an extent to the rise in the number of cases of type 2 diabetes across the African continent [9].

Recent studies in some countries have identified modifiable factors that influence body fat levels in children and adolescents to various extents, including increased physical activity and sedentary time [10], increased fruit and vegetable consumption [11], high frequency of snacking [12], parental socioeconomic status [4], and skipping of breakfast [13, 14]. The extents to which these variables influence body fat levels of children in our setting are still to be fully exploited. While some studies on overweight/obesity in school-age children have used more accurate methods to assess body fat levels [15], others, especially in developing countries, including Cameroon [4–6], have used BMI, which is a proxy measure of body fatness. Even though BMI is a simple and noninvasive technique and now has internationally accepted cutoff values for children aged 0–5 years [16] and 5–19 years [17], it still has major shortcomings. For instance, BMI does not indicate the distribution of fat in the body and cannot differentiate between lean and fat mass [18]. It has a low sensitivity and can misclassify high numbers of children with excess body fat [19].

The differences in body composition and distribution of body fat have been well established in some ethnic groups. For instance, compared with Caucasians, Asians tend to accumulate more body fat at an equivalent BMI value and have a reduced amount of fat-free mass. Also, the body fat is more centrally than peripherally distributed [20, 21]. This has led to a revision of BMI cutoff points for Asian populations by the WHO [22]. However, such comparisons that include African countries are not available because of limited data on the assessment of body fatness using other simple (skinfold thickness and waist circumference) and two-compartment (bioelectrical impedance analysis (BIA) and dual energy X-ray absorptiometry (DXA)) techniques, which allow for a more accurate assessment of the distribution of body fat as well as distinguishing between fat mass and fat-free mass.

BIA is a simple, portable, and an inexpensive technique used for the assessment of body fat that requires less training [23]. A previous study that included a multiethnic group of adolescents had indicated that there were no significant differences in mean percentage body fat (%BF) estimates using DXA and a BIA instrument [24]. Hence, BIA can be used as an appropriate technique in estimating %BF in children and adolescents as suggested in a recent review [25]. The use of BIA in African settings to assess body fat is limited. However, it has been used in assessing fat-free mass in populations of African origin in which there was high agreement between the values determined using isotope dilution and BIA equations [26].

This study aims to assess the relationships between selected behavioral factors and percentage body fat estimated using BIA in primary school children living in urban settings of two administrative regions of Cameroon.

## 2. Materials and Methods

### 2.1. Participants

This cross-sectional analysis included data from apparently healthy school-age children (6–11 years) living in two urban settings; Bamenda and Bafoussam, which are the headquarters of the North West and Western Regions of Cameroon, respectively. Four schools (2 public and 2 private) were randomly selected in each of the urban settings (8 schools in total) from established lists of primary schools obtained from the delegations of basic education in both regions, which constituted the sampling frame. The selected schools were visited to present the research objectives and to give the consent information to the head teachers. The schools that declined from participating were immediately replaced by another randomly selected school. Also, in order to maximize the age distribution of the sample, we randomly selected 50% of children in each of the six classes of the different schools. Through this exercise, 1412 pupils were randomly selected. In addition, the selected children were given envelopes containing the consent information, study objectives and the questionnaire to take to their parents/guardians. 1106 questionnaires were returned, giving a response rate of 78.3%. Furthermore, participants with missing information were excluded from the analysis (*n*=98), leaving a final study population of 1008 pupils.

### 2.2. Ethical Considerations

Ethical clearance for this study was obtained from the Institutional Review Board of the Catholic University of Cameroon. Administrative clearances were obtained from the regional delegations for basic education of the North West and Western Regions. All parents/guardians and head teachers gave written informed consent before the study started. Verbal assent was also obtained from each child before measurements were carried out.

## 3. Data Collection

### 3.1. Anthropometry and Percentage Body Fat Measurements

Measurements were carried out during a second visit to the schools during morning hours by trained personnel. All participants had no shoes and were in light clothing during anthropometric measurements. Standing height to the nearest 0.1 cm was measured using a portable stadiometer (Seca 213, Hamburg, Germany). Body weight was also measured to the nearest 0.1 kg using a digital scale/body composition monitor (Omron BF511, Kyoto, Japan). BMI was calculated as weight (kg) divided by the square of the height (m). Z-scores for BMI were calculated using the WHO growth reference for children aged 5 to 19 years [17] and used to categorize participants as underweight, healthy weight, overweight, or obese.

Percentage body fat (%BF) was measured by whole-body BIA to the nearest 0.1% using a digital scale/body composition monitor (Omron BF 511, 50 kHz, 500 *µ*A, Kyoto, Japan), which includes an 8-sensor technology using both hands and feet. The participants stood with bare feet on electrodes on the scale with their knees and back straight while grasping a handle that also includes electrodes with both their hands horizontally raised, elbows extended straight, and maintaining a 90°-angle to the body. A previous study that compared body composition estimates using BIA devices with DXA and whole body magnetic resonance imaging indicated that the use of devices with additional hand electrodes provides a more accurate prediction of body composition and are suitable for public use [27]. In order to determine percentage body fat, the device uses electrical impedance, along with the participant's height, weight, age, and gender to generate results. The readings were obtained in duplicates and the average recorded. According to the manufacturer's instructions, percentage body fat was measured two hours or more after breakfast. Omron Healthcare references for age and gender were used to classify the study participants into low, normal, high, and very high %BF [28].

### 3.2. Parent Questionnaire

The information on the different behavioral factors that influence body fat levels in children were reported by their parents using a pretested questionnaire, which was developed in both English and French. The section of the questionnaire on eating habits included questions on the frequency of consumption of fruits and vegetables, breakfast, and snacks. The parents also reported the out-of-school physical activity and sedentary lifestyle of the children and provided information on their education level as explained below.

#### 3.2.1. Eating Habits

The study participants were classified according to their parents' responses on the frequency of fruit and vegetable consumption as follows: never, occasionally, and everyday. Also, breakfast consumption was categorized as follows: skip/rarely, 1–2 times/week, 3–4 times/week, and daily.

In the absence of a validated food frequency questionnaire in our setting, parents were asked to select the frequency of consumption of the following snack foods (consumed in-between meals) by their children as follows: biscuits, popcorn, sweets, yogurt, cakes, sugar-sweetened beverages, crackers, and bread. The frequency of snack consumption was categorized as follows: once a day, twice a day, three times a day, and greater than three times a day.

#### 3.2.2. Out-of-School Physical Activity and Sedentary Lifestyle

This study used a set of activities to assess the level of physical activity in the children, which included the following: football, handball, jogging, hopscotch, household chores, skipping rope, dancing, farm work, walking to school, cycling, hide and seek, and playing on the playground. The parents selected the weekly (Monday to Sunday) participation of their children in the above activities from seven categories, which ranged from a minimum duration of one time/week to a maximum of 7 times/week. The mean time of involvement in the above activities was then calculated, and participants were grouped according to their physical activity levels as follows: low (≤2 times/week), moderate (>2 to 4 times/week), and high (>4 to 7 times/week) [29].

The activities used to assess out-of-school sedentary lifestyle included screen-based sedentary behaviors: playing video games, watching TV, and using the computer. Similarly, the parents selected the hourly involvement of their children in the above activities from six categories, which ranged from 1 hour/day to 6 hours/day during the week (weekdays and weekend days). The average time (in hours/day) spent in the above activities was also calculated and used to assign the children into the following groups of sedentary lifestyle as follows: low (≤one hour/day), moderate (>1 to 3 hours/day), and high (>3 to 6 hours/day) [30].

It is worth mentioning that we do not have a valid instrument to assess physical activity and sedentary lifestyle of school-age children in our setting and did not carry out any validity test. However, physical activity and sedentary lifestyle sections of our questionnaire were similar to that of a previous report [31].

### 3.3. Motorization to School

The parents indicated with a “yes” or “no” whether the children are dropped at school using their private cars, taxis, motorbikes, or use the school buses.

### 3.4. Parental Education

The parents selected the number of years spent in education activities from the following categories: none, ≤6 years, 7 to 13 years, and >13 years of education. They were then assigned to their corresponding educational levels as follows: no education, primary, secondary, and tertiary levels. The children were assigned to the appropriate group using the parent with the highest level of education.

The regional delegations for basic education and the school administration provided information on the type of school (public or private).

### 3.5. Statistical Analysis

Data analysis was carried out using the statistical software, R version 3.4.1, which includes the “QUANTREG” package [32]. Normality of continuous variables was checked using the Kolmogorov–Smirnov (K-S) test. Body weight, height, and BMI were adjusted for age and gender using the WHO growth monitoring software (AnthroPlus) for children between the ages of 5 and 19 years [17]. Also, the Student's *t*-test for independent samples and the Mann–Whitney *U* test were used for the comparison of means of continuous variables by gender, and values have been reported as mean (minimum–maximum). The chi-squared test was used for comparison of proportions, and we have reported the findings as frequencies and percentages.

In addition, the differences in median percentage body fat were compared across selected factors by using the Kruskal–Wallis test (unadjusted analysis).

Further, the association between %BF and selected factors was evaluated using multivariable quantile regression analysis. In this adjusted analysis, %BF was modeled as the dependent variable. The median was modeled rather than the mean because %BF did not meet the distributional assumption of normality. Moreover, the findings are easy to understand because the regression estimates indicate the difference in median %BF associated with each factor with respect to a reference group. In the regression analysis, the model was adjusted for design variables (class, school, and region), age, gender, and all variables in the unadjusted analysis. The regression coefficient estimates of the median %BF and corresponding standard errors were calculated using the Powell Kernel approach [32]. Statistical significance was considered at *p* < 0.05 level.

## 4. Results

This study included a sample of 1008 school-age children with a mean age of 8.9 ± 1.6 years (Table 1). According to the WHO criteria, 15.9% of the study population was overweight/obese (BMI). Also, the overall prevalence of overweight/obesity (%BF) was 20.5% using the Omron Healthcare definition. Table 1 also indicates that the mean BMI standard deviation score (SDS) was significantly (*p* < 0.001) higher for boys than girls and mean %BF was significantly (*p* < 0.001) higher for girls than boys. This is also reflected in the distribution of the study participants into the overweight/obese (BMI) and overweight/obese (%BF) groups. More boys (16.2%) were overweight/obese (BMI) compared to girls (15.6%). On the contrary, more girls (22.6%) were overweight/obese (%BF) compared to boys (18.5%). Nevertheless, these differences were not statistically significant. Private schools had a higher prevalence of overweight/obesity (%BF) (24.2%) compared to public schools (22.5%). However, this difference was also not statistically significant (*p*=0.165). The majority of the children in both public (59.1%) and private (58.2%) schools had normal body fat levels (%BF). There were no statistical differences in anthropometric variables by region.

Table 2 shows a comparison of selected behavioral factors between boys and girls. More than a third (40.2%) of the study participants never eat fruits and vegetables. 16.6% of the study participants skip/rarely had breakfast. Out of the total number of those who skip/rarely had breakfast, 82.1% were between the ages of 8 and 11 years. Only 31.3% of the study participants had breakfast daily. Also, more than half (68.4%) of the children had snacks (in-between meals) ≥3 times/day with a higher proportion of girls having snacks greater than three times a day. In addition, half (50.9%) of the study participants had a low out-of-school physical activity level (≤2 times/week). There was no significant difference in out-of-school physical activity between boys and girls (*p*=0.167). Out-of-school sedentary lifestyle was significantly different between boys and girls (*p*=0.002) with a third of girls having a high out-of-school sedentary lifestyle (>3–6 hours/day). 52.7% of the children indicated they use motorization to school.

Table 3 shows that median %BF was comparable across the selected factors. The median %BF was significantly (*p* < 0.001) higher for participants who never eat fruits and vegetables, skip/rarely have breakfast, consume snacks (in-between meals) more than three times a day, have a low out-of-school physical activity level, and have a high out-of-school sedentary lifestyle compared to those who eat fruits and vegetables daily, take breakfast daily, have snacks in-between meals once a day, have a high out-of-school physical activity level, and have a low out-of-school sedentary lifestyle. A similar trend was observed on a gender basis with girls having higher median %BF values across the different factors.

Never consuming fruits and vegetables and consuming snacks (in-between meals) more than three times a day were the two factors that contributed significantly (*p* < 0.001) to the highest median %BF when the full sample was considered. Children who never eat fruits and vegetables and consumed snacks (in-between meals) had the highest median %BF of 25.7% compared to 17.1% for those who eat fruits and vegetables daily and those who consume snacks (in-between meals) once a day. When the data were split by gender, skipping/rarely having breakfast also contributed significantly (*p* < 0.001) to the highest median %BF in girls. There was a 10% difference in median %BF between girls who skip/rarely have breakfast and those who have breakfast daily. In both boys and girls, the use of motorization to school resulted in a significantly (*p* < 0.001) higher median %BF when compared to those who did not use motorization to school.

The multiple quantile regression estimates of the median %BF are presented in Table 4. The daily consumption of fruits and vegetables (*p* < 0.001), daily breakfast consumption (*p* < 0.001), and high out-of-school physical activity (>4–7 times/week) (*p* < 0.001) were significantly and negatively associated with %BF. On the contrary, there was a significant positive association between consumption of snacks (in-between meals) more than three times a day (*p* < 0.001), high out-of-school sedentary lifestyle (>3–6 hours/day), and motorization to school (*p*=0.005) with %BF.

When the full sample was considered, the two factors with the largest relationship with %BF were the consumption of fruits and vegetables and the consumption of snacks (in-between meals). The study participants who ate fruits and vegetables daily had a 4.95% lower median %BF compared with those who never ate fruits and vegetables (*p* < 0.001). Also, the median %BF of those who consume snacks (in-between meals) more than three times a day was 3.69% higher than those who consume snacks once in a day (*p* < 0.001). These two variables also contributed to similar changes in median %BF in boys. Among girls, the consumption of snacks (in-between meals) more than three times a day, the daily consumption of breakfast, high out-of-school physical activity (>4–7 times/week) and high out-of-school sedentary lifestyle (>3–6 hours/day) largely contributed to changes in %BF. For instance, the median %BF of girls who consumed snacks (in-between meals) more than three times a day was 5.32% higher than their peers who had snacks once in a day (*p* < 0.001). Also, the girls who have breakfast daily had a 5.42% lower median %BF compared with those who skip/rarely have breakfast (*p* < 0.001). In addition, girls with a high out-of-school physical activity (>4–7 times/week) had a 6.55% lower median %BF compared with those who had a low out-of-school physical activity (≤2 times/week) (*p* < 0.001).

## 5. Discussion

This study is aimed at evaluating the associations between selected behavioral factors and %BF in urban school-age children. It provides data on %BF of school children assessed using BIA for the first time in Cameroon. This study has confirmed (after adjusting for different variables) that eating fruits and vegetables every day, having breakfast >3 times/week, and physical activity >2 times/week were inversely associated with %BF.

Eating fruits and vegetables every day was significantly associated with lower median %BF because fruits and vegetables have a low energy density, hence a small amount of calorie consumption. A previous study had indicated that participants who had a low fruit and vegetable intake had higher odds for obesity compared with their counterparts with a higher fruit and vegetable consumption [33]. However, a recent systematic review and meta-analysis indicated that there was no significant difference in daily energy intake between subjects with low and high fruit and vegetable consumption [11].

Having breakfast >3 times/week was significantly associated with a lower %BF, and breakfast skippers had a higher median %BF compared to those with high frequency of breakfast consumption. Similar findings have been observed in different reports. For instance, a study revealed that children who had breakfast at least 5 times/week had a significantly lower %BF compared to the less frequent breakfast consumers [14]. Also, recent studies have confirmed that breakfast eaters had a significantly lower intake of total fat compared to their counterparts who skip breakfast [34] and that skipping breakfast at the age of 4 years was associated with a higher %BF at the age of 6 years [35]. Some studies have suggested that the higher levels of %BF observed in breakfast skippers could be explained by a positive energy balance and weight gain, which result from an increased consumption of unhealthy snacks during the day [36] and late night eating among breakfast skippers [37]. Apart from skipping breakfast, it is also possible that the types of food eaten during breakfast could be contributing to higher adiposity levels [38]. However, a recent systematic review revealed that the evidence relating dietary factors and obesity markers is inconclusive [39].

This present study has also confirmed that the consumption of snacks (in-between meals) greater than three times a day, high out-of-school sedentary activity (>3–6 hours/day), and motorization to school were positively associated with %BF.

The consumption of snacks (in-between meals) greater than three times a day contributed to higher levels of median %BF. This is consistent with findings from recent studies, which reported that high snack frequency was associated with higher risk of central obesity in U.S. children [40] and BMI-defined overweight/obesity in 11- to 13-year-old Italian adolescents [41]. This observation could be due to the inability of high frequency snackers to compensate for the excess energy obtained from high sugar and fat-containing snacks by consuming less calories in their subsequent meals [42]. However, in another report, no association was observed between snack frequency and changes in BMI *z*-score [43]. The absence of a universally acceptable definition of what constitutes a snack could explain these inconsistencies. The constituents of snack food used in our study were similar to those of a study by Bo et al. [41]. Also, the timing of snack consumption and overweight/obesity could explain these differences. For instance, Bo et al. [41] recorded the highest prevalence of overweight/obesity among study participants who snack in the evening after dinner compared with those who snack earlier.

Physical activity >2 times/week was associated with lower %BF. It is well known that when physical activity levels increase, there is more energy expenditure, which lowers the risk of overweight/obesity [44], and this is beneficial to children. However, no association was observed between physical activity and BMI-defined obesity in a sample of Nigerian children [45]. This could have been as a result of the small number of obese children that were included in the Nigerian study. In addition, inconsistent findings on the association between physical activity and adiposity have been reported in systematic reviews (that included cohort studies), which indicate either a strong or weak association [46, 47].

A high out-of-school sedentary lifestyle was significantly positively associated with higher levels of %BF. This is consistent with a recent report, which revealed that sedentary time (assessed using parent-reported computer games use and TV viewing) was positively associated with %BF and BMI in crude models [48]. Another study indicated similar relationships between objectively measured sedentary time and %BF [10]. A systematic review has also indicated that higher levels of sedentary time were associated with unfavorable body composition [49]. However, a study in Kenya did not record an association between sedentary time and %BF [50]. Even though the Kenyan study was similar to ours in the sense that assessment of sedentary lifestyle did not include activities in school, the sedentary time was self-reported by the children with the possibility of recall bias. In our study, this bias was limited by asking only parents (who have a higher ability to recall and are most familiar to the children) to report the sedentary lifestyle of their children.

Motorization to school was significantly positively associated with higher %BF levels. This could be explained by the fact that children who use motorization to school have a lower energy expenditure compared to those who walk to school. In this present study, children in private schools had a slightly higher level of median %BF compared to those in public schools. However, this was not statistically significant after adjusting for age, gender, design variables, and all variables in the unadjusted analysis. A report had indicated that children in private schools tend to have a higher sedentary time [50] and higher odds for overweight/obesity [51] compared to their peers in public schools. The high sedentary time could contribute to an extent to the higher levels of %BF observed in children in private schools in our study. Parental level of education was not associated with %BF. This is consistent with a recent report on BMI-defined overweight/obesity in Brazilian children [52].

The findings of this present study need to be interpreted with caution. First, causal inference cannot be established using a cross sectional design and the study sample is not nationally representative. The sample included children from only two out of the ten administrative regions of the country. It was also not possible to control for tanner stage. A study had revealed that children in private schools had a higher risk of not meeting the guidelines of physical activity compared to those in public schools [52]. During the study period, we observed that more than half of the private schools have facilities that promote a sedentary lifestyle while in school like TV sets, and board games, which children use during their play time. We did not investigate the contribution of these aspects to differences in %BF.

Even though BIA is simple, portable, noninvasive, quick and inexpensive [53], there is evidence that the validity of impedance readings may be affected by the water content of the body. For instance, a study indicated that increased total body water (TBW) results in an underprediction of %BF in obese subjects [54]. In our study, it was not possible to standardize breakfast consumption. While some children might have eaten a lot for breakfast, others may not have eaten anything. Thus the fasting time, which influences TBW, could not have been uniform across the sample. Also, BIA has been found to be population-specific [18], and it is still unclear whether the instrument used to assess %BF in this study is suitable for an African population.

Despite the above limitations, we have been able to quantify the differences in median %BF across different factors after adjusting for different variables using multiple quantile regression analysis. Even though proxy measures were used to estimate physical activity and sedentary lifestyle in this study, these measures provide information on the type of activity and could compliment objective measurements in children [3].

## 6. Conclusions

Our present study has indicated that the consumption of fruits and vegetables, breakfast consumption, and physical activity is inversely associated with %BF. This study also identified that a large proportion of the children do not eat fruits and vegetables (40.2%) and also have a low physical activity level (50.9%). In order to get a deeper understanding of the influence of these factors on %BF, future studies will need to identify the determinants of low fruit and vegetable intake, low frequency of breakfast consumption, and low physical activity. The consumption of snacks (in-between meals), sedentary lifestyle, and motorization to school are positively associated with %BF. This means promoting a reduction in consumption of snacks, sedentary lifestyle, and encouraging walking to school may reduce %BF in children. Further research needs to be carried out in other regions of Cameroon to clarify the effects of these behavioral factors on %BF and assess whether any observed differences in %BF are of clinical relevance.

## Figures and Tables

**Table 1:** Descriptive characteristics of study population.

Variables	Gender	
	Boys (*N*=507)	Girls (*N*=501)
Age (years)	9.0 (6.0–11.0)	8.9 (6.0–11.0)
Body fat (%)	20.0 (8.5–41.5)^a^	22.8 (8.5–48.5)^a^
Weight-SDS	0.24 (−3.11–3.80)	0.14 (−3.42–3.91)
Height-SDS	0.01 (−3.82–4.22)	0.04 (−4.01–6.22)
BMI-SDS	0.41 (−2.26–3.06)^a^	0.18 (−3.58–3.71)^a^
Weight status		
** **Underweight	4 (0.8)	9 (1.8)
** **Healthy weight	421 (83.0)	414 (82.6)
** **Overweight	74 (14.6)	62 (12.4)
** **Obesity	8 (1.6)	16(3.2)
Percentage body fat		
** **Low	105 (20.7)	77 (15.4)
** **Normal	308 (60.8)	311 (62.1)
** **High	63 (12.4)	71 (14.1)
** **Very high	31 (6.1)	42 (8.4)
Type of school		
** **Public	240 (47.3)	243 (48.5)
** **Private	267 (52.7)	258 (51.5)

For continuous variables, values = mean (minimum–maximum) and for weight status and percentage body fat, values = *n* (%). WHO criteria and Omron Healthcare reference were used to define weight status and percentage body fat level, respectively. ^a^*p* < 0.001.

**Table 2:** Behavioral characteristics of boys and girls.

Factors	Boys, *n* (%)	Girls, *n* (%)	*p* Value
Fruit and vegetable consumption			0.257
** **Never	192 (37.9)	213 (42.5)	
** **Occasionally	162 (32.0)	156 (31.1)	
** **Everyday	153 (30.2)	132 (26.3)	

Breakfast consumption			0.410
** **Skip/rarely	81 (16.0)	86 (17.2)	
** **1–2 times/week	115 (22.7)	123 (24.6)	
** **3–4 times/week	157 (31.0)	131 (26.1)	
** **Daily	154 (30.4)	161 (32.1)	

Snacks (in-between meals)			0.001
** **Once a day	64 (12.6)	57 (11.4)	
** **Twice a day	89 (17.6)	109 (21.8)	
** **Three times a day	167 (32.9)	111 (22.2)	
** **Greater than three times a day	187 (36.9)	224 (44.7)	

Out-of-school physical activity			0.167
** **≤2 times/week	243 (49.7)	270 (53.9)	
** **>2–4 times/week	192 (37.9)	168 (33.5)	
** **>4–7 times/week	72 (14.2)	63 (12.6)	

Out-of-school sedentary lifestyle			0.002
** **≤1 hour/day	194 (38.3)	152 (30.3)	
** **>1–3 hours/day	200 (39.4)	192 (38.3)	
** **>3–6 hours/day	113 (22.3)	157 (31.3)	

Motorization to school			
** **No	246 (48.5)	230 (45.9)	0.406
** **Yes	261 (51.5)	271 (54.1)	

Parental education			0.768
** **No education	98 (19.3)	83 (16.6)	
** **Primary	120 (23.7)	111 (22.2)	
** **Secondary	155 (30.6)	148 (29.5)	
** **Higher education	134 (26.4)	159 (31.7)	

**Table 3:** Median of percentage body fat of school-age children (*N*=1008).

Factors	Full sample		Boys		Girls	
	Median	*p* Value^a^	Median	*p* Value^a^	Median	*p* Value^a^
Fruit and vegetable consumption		<0.001		<0.001		<0.001
** **Never	25.7		22.8		28.5	
** **Occasionally	20.0		18.5		22.8	
** **Everyday	17.1		15.7		18.5	

Breakfast consumption		<0.001		<0.001		<0.001
** **Skip/rarely	25.6		22.8		28.5	
** **1–2 times/week	25.6		24.2		28.5	
** **3–4 times/week	18.5		17.4		18.5	
** **Daily	17.1		15.7		18.5	

Snacks (in-between meals)		<0.001		<0.001		<0.001
** **Once a day	17.1		15.7		17.1	
** **Twice a day	17.1		15.7		18.5	
** **Three times a day	20.0		18.5		22.8	
** **Greater than three times a day	25.7		24.2		28.5	

Out-of-school physical activity		<0.001		<0.001		<0.001
** **≤2 times/week	24.2		22.8		27.0	
** **>2–4 times/week	18.5		17.1		22.4	
** **>4–7 times/week	15.7		15.7		15.7	

Out-of-school sedentary lifestyle		<0.001		<0.001		0.052
** **≤1 hour/day	18.5		15.7		22.8	
** **>1–3 hours/day	22.8		20.0		22.8	
** **>3–6 hours/day	23.5		22.8		24.2	

Motorization to school		<0.001		<0.001		<0.001
** **No	18.5		17.1		20.0	
** **Yes	22.8		21.4		26.0	

Type of school		0.258		0.097		0.886
** **Public	20.0		18.5		22.8	
** **Private	22.8		20.0		22.8	

Parental education		0.152		0.238		0.176
** **No education	20.0		17.1		22.8	
** **Primary	22.6		20.0		22.8	
** **Secondary	22.8		20.0		24.2	
** **Higher education	21.0		20.0		22.8	

^a^Kruskal–Wallis test was used to compare median percentage body fat across all groups.

**Table 4:** Multiple quantile regression coefficients for the association between selected behavioral factors and percentage body fat.

Factors	Full sample			Boys			Girls		
	Estimate	SE	*p* Value	Estimate	SE	*p* Value	Estimate	SE	*p* Value
Intercept	9.01	2.66	0.001	9.12	4.50	0.043	15.26	4.10	<0.001

Fruit and vegetable consumption									
** **Never	0			0			0		
** **Occasionally	−2.62	0.61	<0.001	−2.37	0.94	0.012	−1.80	1.00	0.074
** **Everyday	−4.95	0.67	<0.001	−4.69	1.08	<0.001	−3.39	1.05	0.001

Breakfast consumption									
** **Skip/rarely	0			0			0		
** **1–2 times/week	−0.37	0.76	0.172	−0.01	1.18	1.000	−1.00	1.15	0.382
** **3–4 times/week	−2.11	0.87	0.016	−1.70	1.15	0.142	−4.19	1.44	0.004
** **Daily	−3.29	0.82	<0.001	−2.76	1.10	0.013	−5.42	1.22	<0.001

Snacks (in-between meals)									
** **Once a day	0			0			0		
** **Twice a day	0.23	0.78	0.765	0.40	1.31	0.758	2.19	1.36	0.107
** **Three times a day	2.11	0.86	0.015	3.45	1.30	0.008	2.40	1.55	0.125
** **Greater than three times a day	3.69	0.82	<0.001	4.33	1.28	0.001	5.32	1.47	<0.001

Out-of-school physical activity									
** **≤2 times/week	0			0			0		
** **>2–4 times/week	−2.39	0.58	<0.001	−3.51	1.09	0.002	−1.62	0.85	0.050
** **>4–7 times/week	−3.66	0.81	<0.001	−3.66	0.86	<0.001	−6.55	1.66	<0.001

Out-of-school sedentary lifestyle									
** **≤1 hour/day	0			0			0		
** **>1–3 hours/day	2.22	0.57	<0.001	3.12	0.84	<0.001	2.40	0.91	0.009
** **>3–6 hours/day	3.01	0.68	<0.001	3.83	1.09	<0.001	4.74	1.10	<0.001

Motorization to school									
** **No	0			0			0		
** **Yes	1.39	0.49	0.005	1.22	0.76	0.107	1.20	0.76	0.116

Type of school									
** **Public	0			0			0		
** **Private	0.74	0.61	0.222	0.44	1.04	0.669	0.95	0.99	0.335

Parental education									
** **No education	0			0			0		
** **Primary	0.51	0.79	0.516	0.50	1.22	0.682	0.91	1.22	0.457
** **Secondary	0.49	0.76	0.514	0.27	1.01	0.789	1.87	1.19	0.116
** **Higher education	0.42	0.74	0.567	0.70	1.09	0.523	0.58	1.11	0.598

SE, standard error. Model adjusted for design variables (school, class, and region), age, gender, and all variables in the unadjusted analysis. The estimate associated with each category is the difference in median percentage body fat compared with the reference category.

## Data Availability

The data used to support the findings of this study are available from the corresponding author upon request.

## Supplementary Materials

Supplementary Materials Parent questionnaire.
