# Supplementary material for: A Median Analysis of Factors Influencing Body Fatness in Urban School-Age Children in Cameroon
Source: J Nutr Metab. 2019 Feb 3;2019:1856069. doi: 10.1155/2019/1856069 (PMC6378010; doi:10.1155/2019/1856069)
Supplement: Supplementary Materials — Parent questionnaire. [file 1856069.f1.pdf]

PARENT QUESTIONNAIRE

Please complete every line of this questionnaire which will take approximately 15 minutes.

Sample code

A. Education level

MOTHER	FATHER
Indicate the number of years you have spent in education (please tick)<br>a) None<br>b) ≤ 6 years<br>c) 7 to 13 years<br>d) > 13 years	Indicate the number of years you have spent in education (please tick)<br>a) None<br>b) ≤ 6 years<br>c) 7 to 13 years<br>d) > 13 years

B. Eating habits

Please tick the appropriate frequency of consumption of the following items by your child.

ITEM		FREQUENCY			
**Fruit and vegetable**		Never	Occasionally	Daily	
**Breakfast**		Skip/rarely	1 - 2 times/year	3 - 4 times/week	daily
**Snack foods (in-between meals)**		Once a day	Twice a day	3 times/day	>3 times/day
	Biscuits				
	Popcorn				
	Sweets				
	Yoghurt				
	Cakes				
	Sugar sweetened beverages				
	Crackers				
	Bread				

C. Motorization to school

Do you drop your child in school using any of the following means: private cars, taxis, motorbikes, or school bus? (please tick): a) Yes b) No

D. Out of school physical activity and sedentary lifestyle

Did your CHILD do the following activities in the **past 7 days?** (please circle the right answer)		**MONDAY - FRIDAY**<br>How many times?<br>**Mon – Fri**	**SATURDAY - SUNDAY**<br>How many Times?<br>**Sat – Sun**
EXAMPLE: Bike riding	Yes No	**2**	**1**
**Physical activity**			
Football	Yes No		
Handball	Yes No		
Jogging	Yes No		
Hopscotch	Yes No		
Household chores	Yes No		
Skipping rope	Yes No		
Dancing	Yes No		
Farm work	Yes No		
Walking to school	Yes No		
Cycling	Yes No		
Hide and seek	Yes No		
Playing on the playground	Yes No		
Did your CHILD do the following activities in the **past 7 days?** (please circle the right answer)		**MONDAY - FRIDAY**<br>How many hours?	**SATURDAY – SUNDAY**<br>How many hours?
**Sedentary lifestyle**			
Playing video games	Yes No		
Watching TV	Yes No		
Using the computer	Yes No		

Adapted from the UK children's physical activity questionnaire (C-PAQ). Medical Research Council (MRC), Epidemiology Unite, 2011.
